# Introducing the Forensic Research/Reference on Genetics knowledge base, FROG-kb

**DOI:** 10.1186/2041-2223-3-18

**Published:** 2012-09-01

**Authors:** Haseena Rajeevan, Usha Soundararajan, Andrew J Pakstis, Kenneth K Kidd

**Affiliations:** 1 Department of Genetics, Yale University School of Medicine, 333 Cedar Street, P.O.Box 208005, New Haven, CT 06520-8005, USA; 2 Center for Medical Informatics, Yale University School of Medicine, New Haven, CT, 06520, USA

**Keywords:** Knowledge base, SNP, InDels, Forensics, Individual identification, Ancestry inference markers, Lineage informative markers

## Abstract

**Background:**

Online tools and databases based on multi-allelic short tandem repeat polymorphisms (STRPs) are actively used in forensic teaching, research, and investigations. The Fst value of each CODIS marker tends to be low across the populations of the world and most populations typically have all the common STRP alleles present diminishing the ability of these systems to discriminate ethnicity. Recently, considerable research is being conducted on single nucleotide polymorphisms (SNPs) to be considered for human identification and description. However, online tools and databases that can be used for forensic research and investigation are limited.

**Methods:**

The back end DBMS (Database Management System) for FROG-kb is Oracle version 10. The front end is implemented with specific code using technologies such as Java, Java Servlet, JSP, JQuery, and GoogleCharts.

**Results:**

We present an open access web application, FROG-kb (Forensic Research/Reference on Genetics-knowledge base, http://frog.med.yale.edu), that is useful for teaching and research relevant to forensics and can serve as a tool facilitating forensic practice. The underlying data for FROG-kb are provided by the already extensively used and referenced ALlele FREquency Database, ALFRED (http://alfred.med.yale.edu). In addition to displaying data in an organized manner, computational tools that use the underlying allele frequencies with user-provided data are implemented in FROG-kb. These tools are organized by the different published SNP/marker panels available. This web tool currently has implemented general functions possible for two types of SNP panels, individual identification and ancestry inference, and a prediction function specific to a phenotype informative panel for eye color.

**Conclusion:**

The current online version of FROG-kb already provides new and useful functionality. We expect FROG-kb to grow and expand in capabilities and welcome input from the forensic community in identifying datasets and functionalities that will be most helpful and useful. Thus, the structure and functionality of FROG-kb will be revised in an ongoing process of improvement. This paper describes the state as of early June 2012.

## Background

It is considerably more than a decade since the forensic community settled on a set of short tandem repeat (STR) polymorphisms (hence also STRP) for human identity testing [1]. These markers are multi-allelic and are excellent for individual matching of suspect and crime scene DNA. While 13 STRs form the core of the FBI Laboratory’s CODIS (Combined DNA Index System), the 10 core loci used in the UK and much of Europe consist of eight loci that overlap with CODIS plus seven additional markers that include the five new European Standard Set (ESS) [2]. Discussions on the best options on expanding the core sets of loci are underway [3-5]. Development of reliable commercial multiplex kits tailored specifically for these sets of markers has led to large offender databases and large amounts of allele frequency data accumulated for these markers on a wide range of populations around the world. Online tools and databases have followed to allow users to reference and predict population affiliations: Canadian Random Match Calculator [6] (http://www.csfs.ca/pplus/profiler.htm), European Network of Forensic Science Institute’s DNA WG STR Population Database (http://www.str-base.org/index.php), STRBase (http://www.cstl.nist.gov/strbase/) [7], PopAffiliator (http://cracs.fc.up.pt/popaffiliator/) [8], and pop.STR (http://spsmart.cesga.es/popstr.php) [9]. One argument for continuing use of these tools and marker panels is the large number of individual offender DNA profiles in databases allowing ‘cold hits’, that is, identification of the criminal based on a database match to crime scene DNA. The extensive allele frequency data that have been accumulated over the years in large public databases also allow population-specific estimates of the probability of a random match of two unrelated individuals. However, it is exactly the high level of polymorphism in almost all populations that limits the ability of these markers to determine ancestry of an individual. The large numbers of alleles and high heterozygosity relate to the high mutation rates of these loci; this also means that matching of STRP alleles is matching by state and not of alleles that are identical by descent.

Considering the ease, accuracy, and efficiency in typing single nucleotide polymorphisms (SNPs) and their essentially zero rate of recurrent mutation when compared with STRPs [10-12], SNPs have the potential to be considered for human identification and description in forensic, biomedical, association, as well as epidemiological studies [13-15]. Insertion-deletion polymorphisms (InDels) have most of the desirable characteristics of SNPs and panels have been proposed and have begun to be used in forensics both for individual identification and for ancestry inference [16-18]. However, considerable research is required to establish a reliable set of markers (SNPs or InDels) containing sufficient numbers of markers to provide excellent discriminatory power comparable to or exceeding that of STR markers. Not only do multiple populations need to be studied to identify the best markers but interpretation of results in any application needs the reference allele frequencies in multiple populations. The discriminatory power for individual identification will be population specific and ancestry inference will only be as good as the set of reference populations. Online web tools that demonstrate classification algorithms are being developed for SNP sets as well, ‘The Snipper’ app suite (http://mathgene.usc.es/snipper/) for three ancestry informative AISNP sets (34, 32, and 77 markers) being one of them [19]. Currently, the main limitations of these tools are the number of SNP sets and the range of population data available for computation.

In this paper we introduce FROG-kb (Forensic Resource/Reference on Genetics knowledge base), an open access web tool that allows viewing and retrieval of data as well as calculation of statistics on several forensically relevant SNP sets. FROG-kb’s user interface is versatile in its functionality and comprehensive in the population data available for many SNP sets. The overall goal of FROG-kb is to make allele frequency data for SNPs and other genetic polymorphisms more accessible and useful in a forensic setting. Ancestry, Individual, and Phenotype Informative (AISNP, IISNP, and PISNP) panels [19-24] studied and published from the host lab and elsewhere are currently available in FROG-kb. Each of these panels exists with supporting population data. URL links exist to ALFRED for more details and allele frequency data tables for specific populations not only for the panels themselves but also for each SNP in each panel. Additional information in the underlying ALFRED database and the curated links into other databases make FROG-kb a reference source as well. As frequency data on a population not in the original publication become comprehensively available for the full SNP panel, that population is included in the computations. As new forensically relevant panels of SNPs with meaningful population data are published, they will be systematically added to FROG-kb. The structure and types of contents in FROG-kb are described below followed by descriptions of current functionality with examples.

## Methods

### Database structure

**Figure 1:**
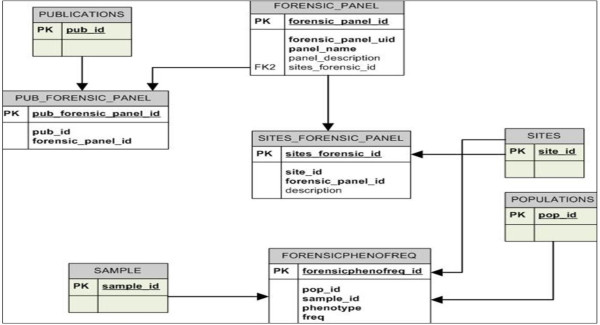
**Additional tables added to ALFRED for FROG-kb.** The full relational schema of ALFRED is described in [26]; these tables and their interconnections illustrate the additions needed for the functionality of FROG-kb.

The underlying data for the FROG-kb implementation are from the allele frequency database ALFRED (http://alfred.med.yale.edu) [25,26]. ALFRED is a relational database and aspects of the structure and relationships of the tables exist in the above mentioned publications. Additional tables accommodate information essential for a human identity testing application. Figure 1 gives the database tables and relationships incorporating only the supplementary tables relevant to FROG-kb. The logic supporting the design of the additional tables and relationships follows. There can be one or many sites (SITES_FORENSIC_PANEL) associated with a defined forensic panel (FORENSIC_PANEL). Every panel is linked to at least one publication (PUB_FORENSIC_PANEL). Such links are clearly identified when the underlying data are unpublished to document the source of the data. The marker phenotype (equivalent to ‘genotype’ based on multiple unavoidable assumptions) frequency for each ‘site - population sample’ combination is pre-calculated and saved in ‘FORENSICPHENOFREQ’. Since these population-marker frequencies do not change for existing data, pre-calculation of the phenotype summary is reasonable and expedites the involved case-specific computations. While all allele frequency data required for running the computations in FROG-kb were already in the ALFRED database, the new tables provide the framework for displaying information related to the different forensically relevant SNP sets in an efficient and user-friendly manner.

### Implementation

The database for FROG-kb is implemented using Oracle version 10 on one of Yale’s institutional database servers where it is maintained. The web front end is built using web developing technologies such as Java, Java Servlet, JSP, JQuery, and GoogleCharts. Almost all of the client-code utilizes JQuery, and the server implementation is in Java. The currently deployed version of FROG-kb has been tested on both PC and Mac, using many different browsers: Mozilla Firefox11.0, Internet Explorer 8.0, Safari 5.1.4, and Google Chrome 17.0 on PC, and Firefox11.0, Internet Explorer 5.0, Safari 5.0 on Mac. We are using Tomcat as our web server which runs on a Windows XP machine.

### Functionalities

**Figure 2:**
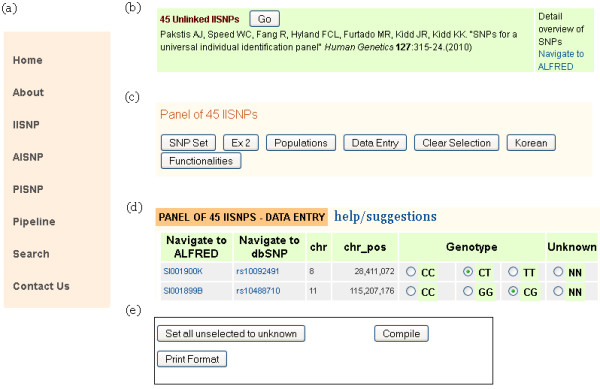
**Snapshot of functionalities on FROG-kb.** Portions of relevant screens seen in FROG-kb. ( **a**) The basic navigation panel seen on the left side of all screens. ( **b**) An example of one of the specific sets of SNPs, in this case an IISNP panel, with the [Go] button to enter the page with options for that set. Also, on the right is the link to ALFRED to view details on all the SNPs in this specific panel. ( **c**) The upper row of buttons for options for a specific panel. Other panels may have a slightly different set and a second row of buttons usually has additional options. However, the list of the SNPs, the list of the populations, the data entry, clearing the data entry fields, and at least one example dataset are common buttons to all panels. ( **d**) The top portion of the data entry page showing the radio buttons to be used to enter the genotype for each SNP. ( **e**) The buttons at the bottom of the data entry screen allowing the option of autofilling for missing data and printing of the input data plus the [Compile] button to initiate calculation.

The user interface layout of FROG-kb is designed to reflect the organization of the contents and functionality, as well as ease of use. Every set of pages relative to a function on FROG-kb originates from a tab on the ‘Main Menu’ (Figure 2A) that appears on the left-hand side of every page. The ‘Home Page’ gives a brief summary of the functions available in FROG-kb and what can be expected soon. The procession through the interface, explained below, is summarized graphically under ‘Pipeline’.

Users can navigate into the functions relevant to each type of SNP panel by selecting the appropriate tab: ‘IISNP’ for Individual Identification SNPs, ‘AISNP’ for Ancestry Inference SNPs and ‘PISNP’ for Phenotype Informative SNPs. Following selection of a particular SNP panel category (IISNP, AISNP, or PISNP), there are multiple published panels listed. The citation information for each of the panels, a ‘Go’ button to navigate into the selected panel, and a ‘Detailed Overview of SNPs’ link to navigate into ALFRED are provided for each (Figure 2B). The link to ALFRED opens the ‘SNP Sets’ page within ALFRED into a new browser window. The SNP Set module in ALFRED has multiple functions, including the ability to see for each SNP a pie chart on Google Maps of frequencies for all populations with data.

Several options are possible after entering a SNP panel page. The functions related to the selected panel are available by selecting the appropriate buttons at the top of the page (Figure 2C). The option SNP Set provides the list of SNPs in the panel. The list includes the dbSNP rs-number of each SNP with an active link to the corresponding dbSNP record for molecular characterization of the SNP. The Populations button provides the list of populations for which comparable calculations can be made. This is the set of populations for which all SNPs in the set have allele frequency data. Conversely, many populations have data on additional SNPs; those SNPs are not included for the calculations. Within the SNP Set functionality in ALFRED additional populations may have data for some, but not all SNPs; those populations are not included in the calculations. Each population name within FROG-kb is an active link to information on the population stored within ALFRED; that page will open in a new browser window. The geographic region of each population is included. A world map in which regions are divided on an arbitrary but convenient basis is available from the link ‘Geographic Region Map’. Example options are also accessible using Ex 1 or similar buttons. These are static screen shots to provide examples.

The most significant interactive function is accessed via Data Entry (Figure 2D) that opens the ability to specify an individual’s multi-site genotype (strictly, phenotype) and then calculate the probability of that genotype in each of the populations. The list of SNPs is sorted by rs-numbers for ease of working with the set. For each SNP on the list the ALFRED UID, dbSNP rs-number, chromosome, and chromosomal position are displayed. The ALFRED UID and rs-number are URL links to ALFRED and to dbSNP SNP information pages, respectively. This is followed by radio buttons for the possible genotypes. The genotype is entered by simply clicking on the radio button for the genotype at each SNP. An obvious assumption is that there is no allele drop out, that is, that a typing result (phenotype) with only one allele detected is really a homozygote. A radio button labeled ‘NN’ is provided for missing data for each SNP, but it is not necessary to click on the ‘NN’ for missing data. For large SNP sets, if the user’s SNP set is also ordered by rs-number in a spreadsheet, selecting the appropriate genotype radio-button should be relatively effortless. (We are aware this input can be tedious; more user friendly options are under development.) At the bottom of the list are three buttons (Figure 2E): Set all unselected to unknown, Print Format, and Compile. The Print Format will generate a condensed version of the input data that can be printed as a permanent record of the input data. The information in the pop-up window can also be copied and pasted into a text editor which in turn can be opened in an Excel spreadsheet. The Compile will initiate calculation and display the results. If there are SNPs with no selection, a warning will be sent with the missing data rows highlighted for easy detection and the option exists to examine which SNPs have no entry and to either enter a genotype or use the Set all unselected to unknown to fill those with ‘NN’. Afterward, it is necessary to click on Compile again.

The calculations are essentially identical for all the IISNP and AISNP panels, just different loci (SNPs) and different interpretations are involved. All SNPs are considered statistically independent at the population level and so the probability of the input multisite genotype is simply the product of the probabilities of the genotypes of the individual loci--the ‘product rule’ in forensics. This calculation is done separately for each population using the allele frequencies estimated for that population and assuming Hardy-Weinberg ratios to estimate the population-specific genotype probabilities. The calculation uses all loci in the panel for which a genotype was entered. A missing genotype, NN in the input, means that locus is skipped for all populations. Only populations with data for all SNPs in the panel are considered in the calculation. Thus, the resulting probabilities/likelihoods that are displayed are based on the same set of loci for all populations.

Special consideration is needed for those situations in which one allele is not observed in a population and hence the observed allele frequency is zero and two genotypes are strictly estimated to be zero. This is especially important for AISNPs since many of the loci are fixed in some populations, but even a SNP in an IISNP panel may not be seen in an isolated population. Given the sample sizes involved, very low frequencies of the ‘missing’ allele cannot be excluded and using a value of zero in the calculations would be incorrect. The approach used is to simply use a very small allele frequency instead of the zero that is the allele frequency present in ALFRED. If one assumes a heterozygote might be seen in the next individual sampled from the population, the allele frequency would then be 1/(2n + 2) where n is the original sample size in which the allele was not seen. Other approaches to the problem can exist. Because of different finite sample sizes and the unavoidable possibility of typing error, an absolute small frequency could be used. Another alternative is to simply not include that locus in any calculations involving a population in which one allele is not seen in the reference sample. This last completely circumvents a fixed locus having an undue influence on population rankings for AISNPs, but also overcompensates when those alleles are seen in the focus genotype. Those and other options can be considered by the community for future implementation as alternatives.

**Figure 3:**
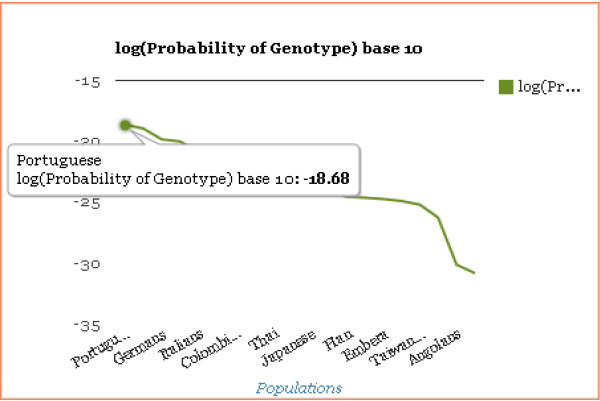
**Graph of log**_**10 **_**(Probability of Genotype).** An example of the plot of results for one individual input genotype with the populations ordered by the log10 probabilities from largest to smallest. This is part of the results display common to all IISNP and AISNP panel calculations. One can mouse over the graph to see the specific population and its value, as illustrated for Portuguese in this example. Not shown here is the list of those populations with the values also displayed on the same page. The option exists to print the list.

The time required to compute the probability of the genotype in each population depends on the number of SNPs in the panel and the number of populations for which the calculations are performed. The results page has a table with the probability values against each population name and a graph of log_10_ (Probability of Genotype) displayed side-by-side. The number of SNPs used in the computation is given. The button View SNPs Used gives the list of SNPs the computation was based on. Print Table Format generates a printable format of the result. The line graph of log_10_ (Probability of Genotype) is drawn utilizing the Google-Chart tools. The population name and the corresponding value are displayed when the mouse is hovered over a point (Figure 3). The geographic region displayed adjacent to a population name can be verified using the image at Geographic Region Map.

There are also buttons with population names, for example Hungarian or Korean that will open a pre-entered data entry page for one individual from the specified population. These can be used in an education mode to explore the dependence of the results on particular SNPs and on the total number of SNPs with data.

The above mentioned functionalities are common to all of the IISNP and AISNP panels. The interpretations of the values calculated differ between the IISNP and AISNP panels (see below). We note that, since the calculations are based on only the SNPs with specified genotypes, it is not necessary that an entire panel be genotyped. Indeed, if by happenstance a user has genotyped SNPs that are in a panel, those SNPs will yield a valid result though there may be little discrimination if only a few SNPs are used.

The single PISNP panel differs in that calculations are not dependent on population but only on the genotype entered. The Irisplex panel for eye color prediction under PISNP has the SNP Set, Data Entry and four pre-entered data entry pages. The eye color prediction computation uses the formula provided in the publication [21] and is given on the web site.

## Results and Discussion

### Interpretation of the calculations

For both the IISNP and AISNP input genotypes the programs currently calculate for each specific population the probability of that multi-locus genotype as the simple product across loci of the frequencies of each specific genotype in the specific population. That simple product is the one displayed for the population in the rank-ordered list and in the graph. The interpretation is different for the two types of SNP panels.

For IISNP panels the ‘Probability of Genotype’ can be interpreted as the match probability, that is, the probability of finding another unrelated individual in the population with the same multilocus genotype. One assumption is infinite population size with no adjustment for finite size or the statistical uncertainty of the allele frequency estimates for each population other than the ‘zero allele frequency’ correction noted above. Another assumption is of statistically independent loci, that is, no linkage disequilibrium. This second assumption has been tested, and satisfied, by analyses in the studies that developed and published the panels. The population-specific values are given in the table ordered from highest to lowest probability, but is easier to visualize in the figure as the orders of magnitude showing how sensitive the match probability estimates are to the population from which the DNA sample was obtained. Of particular note is that the top population gives the highest match probability globally, to the extent the populations represent the global variation. Note that these values are acceptable estimates for whatever data were used. If there were many loci with missing data, the probabilities will be less definitive but will still be the best estimates for the loci used.

For AISNP panels the same calculation is made, simple multiplication of the probability of the population-specific genotypes at the several loci. For AISNPs, however, the interpretation of the probability of the genotype given a population can be considered as proportional to the likelihood of the population given the genotype. As likelihoods, the absolute values have no strict interpretation, only the relative values are interpretable. Thus, the rank orders are meaningful with populations having larger probabilities being more likely origins of the individual DNA profile than populations with smaller probabilities. The important question with respect to ancestry inference is how different the likelihoods are. One rough rule of thumb that could be applied is that likelihoods different by less than an order of magnitude are not significantly different. Thus, while the population with the highest likelihood is the most likely origin of the input genotype, it is not necessarily the correct origin and those with similar, albeit lower likelihoods, cannot be excluded. Indeed, even those more than an order of magnitude less likely are not ‘excluded’, just much less likely.

The likelihoods calculated are the maximum likelihood estimates because they are based on the maximum likelihood estimates for allele frequencies for independent loci with the necessary assumption of Hardy-Weinberg proportions for the genotypes, albeit with a correction for sites with a zero allele frequency. However, those allele frequency estimates have associated standard errors that vary inversely as the sample sizes. Thus, there are greater uncertainties for likelihoods calculated for populations with small sample sizes. Hence, rankings among populations with very similar likelihoods could be different when one or more is based on a very small sample size. The statistical issues with determining significance of an estimated rank order of populations, taking into account all the individual components of uncertainty, are not simple and we currently have no rigorous statistical method identified and implemented. We are not aware of any of the existing AISNP estimation procedures that has solved this problem. We emphasize that users must exercise judgment by recognizing the inherent uncertainty and considering the differences in sample sizes (displayed with each sample name).

### Current panels and examples

Two different Individual Identification SNP panels (IISNPs) provide examples and the ability to calculate match probabilities for user-specified genotypes in each of many populations that have allele frequencies available for all SNPs in the panel. The two panels are (A) 45 unlinked IISNPs from the host lab [20] and (B) the SNPforID 52-plex [21]. Similarly, for Ancestry Inference SNPs (AISNPs), this pilot implementation provides examples and ability to calculate relative likelihoods of ancestry from different populations for user-specified genotypes. FROG-kb has already implemented three different AISNP panels. One is a provisional unpublished panel of 39 SNPs assembled specifically to test functionality of this web application. We have included an illustration of the STRUCTURE output for these AISNPs to document their validity. A set of 128 AISNPs [22,23] and the SNPforID panel of 34 SNPs [19] are two additional panels for which examples and calculations are available. For Phenotype Informative SNPs (PISNPs) we provide a panel of six SNPs for eye color prediction (IrisPlex) along with ability to specify an individual’s genotype and predict eye color from that [24].

### Limitations

FROG-kb can only provide information for the populations comprehensively tested for the entire set of SNPs. For ancestry inference FROG-kb is currently designed for individuals whose ancestry is overwhelmingly from one population or set of closely related populations. Admixed individuals, in the sense of recent ancestors from geographically and genetically different populations, will not necessarily provide meaningful results. For example, one African American genotype gave Ethiopian as the most likely ancestry, understandable because the Ethiopians have allele frequencies at many loci that are intermediate between those of West African and European populations. Other African American genotypes have given West African populations as most likely. Obviously, different African Americans have different combinations of the African and European alleles for the particular AISNPs in a panel. Thus, results for individuals of recent admixed ancestry will be specific to the individual and the AISNP panel.

Currently the allele designations for the genotypes listed on the data entry forms are not consistent with any single standard. Since most SNPs are unambiguous with respect to the strand being called, the user should have no problem making the necessary conversion from the typing data to the genotype codes on the input screen. However, G/C and A/T SNPs need to be specified as to the strand being called; the future standard for FROG-kb will be that the positive strand (pter to qter, 5’ to 3’) will be the reference even for SNPs in genes that are coded on the reverse strand.

Only a small selection of the SNP and InDel panels that have been published is currently available for use in FROG-kb. The initial effort in developing this resource for the forensic community has necessarily focused on the database structure and the website interface. Several other panels of markers have already been identified and work has begun to curate the data and make the material accessible and useful via FROG-kb. We also expect the forensic and research communities to help us identify data that should be included but is not.

## Conclusion

While FROG-kb is a work in progress, the current version of FROG-kb already provides new and useful functionality. We expect FROG-kb to grow and expand in capabilities over the next several months. Indeed, by the time this initial announcement reaches official publication there will likely be changes. We hope that input from the forensic community will help identify those functionalities that are most helpful and useful. We expect FROG-kb to be a useful reference and resource on use of SNP panels in forensics.

## Abbreviations

AISNP: Ancestry informative SNP; App: Application; CODIS: Combined Data Index System; FBI: Federal Bureau of Investigation; IISNP: Individual identification SNP; InDels: Insertion-deletions; KB: Knowledge base; PISNP: Phenotype informative SNP; SNPs: Single nucleotide polymorphisms; STR: Short tandem repeat; STRP: Short tandem repeat polymorphism.

## Competing interests

The authors declare that they have no competing interests.

## Authors’ contributions

HR and KKK are primarily responsible for writing the manuscript. All authors have contributed with suggestions and revisions and have approved the final version.

## Electronic sites referenced

FROG-kb[http://frog.med.yale.edu]

ALFRED[http://alfred.med.yale.edu]

Canadian Random Match Calculator[http://www.csfs.ca/pplus/profiler.htm]

DNA WG STR Population Database[http://www.str-base.org/index.php]

PopAffiliator[http://cracs.fc.up.pt/popaffiliator/]

pop.STR[http://spsmart.cesga.es/popstr.php]

STRBase[http://www.cstl.nist.gov/strbase/]

The Snipper app suite [http://mathgene.usc.es/snipper/]
